# Investigating the linkage between professional development and mathematics instructors’ use of teaching practices using the theory of planned behavior

**DOI:** 10.1371/journal.pone.0267097

**Published:** 2022-04-15

**Authors:** Tim Archie, Charles N. Hayward, Stan Yoshinobu, Sandra L. Laursen

**Affiliations:** 1 University of Colorado Boulder, Boulder, Colorado, United States of America; 2 Department of Mathematics, University of Toronto, Ontario, Canada; The Education University of Hong Kong, HONG KONG

## Abstract

Professional development has been identified as an effective way to increase college STEM instructors’ use of research-based instructional strategies (RBIS) known to benefit student learning and persistence in STEM. Yet only a few studies relate professional development experiences to later teaching behaviors of higher education instructors. This study of 361 undergraduate mathematics instructors, all of whom participated in multi-day, discipline-based workshops on teaching held in 2010–2019, examined the relationship between such participation and later use of RBIS. We found that instructors’ RBIS attitudes, knowledge, and skills strengthened after participating in professional development, and their self-reported use of RBIS became more frequent in the first year after the workshop. Applying the Theory of Planned Behavior as a conceptual framework, we used a structural equation model to test whether this theory could explain the roles of workshop participation and other personal, professional and contextual factors in fostering RBIS use. Findings indicated that, along with workshop participation, prior RBIS experience, class size, and course coordination affected RBIS use. That is, both targeted professional development and elements of the local context for implementation were important in supporting instructors’ uptake of RBIS—but, remarkably, both immediate and longer-term outcomes of professional development did not depend on other individual or institutional characteristics. In this study, the large sample size, longitudinal measurement approach, and consistency of the form and quality of professional development make it possible to distinguish the importance of multiple possible influences on instructors’ uptake of RBIS. We discuss implications for professional development and for institutional structures that support instructors as they apply what they learned, and we offer suggestions for the use of theory in future research on this topic.

## Introduction

Student-centered, research-based instructional strategies (RBIS) have been well established as ways to improve student learning and academic success in US undergraduate STEM education [1,2]. Additionally, RBIS have been shown to lead to more equitable outcomes for students historically underrepresented within STEM [1,3–6]. However, a limited number of instructors use these methods extensively in STEM disciplines, approximately 20% by some estimates [7,8]. As a result, most students do not experience RBIS in the classroom and therefore do not benefit from the associated outcomes.

Professional development has been identified by experts as one of the most influential factors to increase instructor use of RBIS in undergraduate STEM [9–11]. But few studies have demonstrated associations between teaching-focused professional development (PD) and teaching behaviors of STEM college instructors in range of STEM disciplines including chemistry, physics, and biology [12–16]. This study further contributes to this literature by investigating the linkage between professional development and mathematics instructors’ use of RBIS, in this case a particular approach known in mathematics as inquiry-based learning (IBL).

Other factors besides PD have also shown to affect STEM instructors’ RBIS use. Some commonly identified factors that may function as either barriers or supports to RBIS use include: Course content coverage [16–18], time constraints [19–21], student resistance [16,21–23], departmental culture and collegial support [16,24–28], lack of confidence in RBIS or negative attitude towards RBIS [17,24], and class size [22,29]. Less clear are the relationships between PD participation and these other supports and barriers to RBIS use, or their relative importance.

Further, explorations of these relationships should not be haphazard, but instead examined in the context of an appropriate change theory that can accommodate and explain how these salient factors relate to RBIS use. Recent work has called for the use of common change theories in undergraduate STEM education research. Use of common change theories can help to explain how change occurs and can help to advance knowledge outside of discipline specific educational research contexts and helps to make findings more generalizable and transferrable across STEM disciplines [30].

This study applies a well-established social psychology change theory to explain how professional development and other factors work together in shaping instructor adoption of RBIS. While prior literature has begun to draw connections between PD and teaching behavior, few studies have utilized a change theory to explain this connection. In addition to linking PD participation to instructor teaching practices, another purpose of this study was to determine the degree to which a change theory, the theory of planned behavior, can explain instructors’ use of RBIS after participating in professional development.

### Theoretical framework

The theory of planned behavior (TPB) is a socio-cognitive theory that has been applied across a wide variety of disciplines and contexts to explain human behavior [31,32]. The theory has been applied to professional development [33]and teaching practice [34,35], and in contexts that included both professional development and teaching practice [14,36]. The TPB has been identified as a change theory that is broadly applicable to studying change in teaching and learning in STEM [30]. The TPB has been used as a change theory to explain STEM instructors’ use of RBIS following professional development [14,37]. In this study, we apply the TPB to explain how participation in an intensive professional development workshop influences instructors’ use of IBL methods.

**Fig 1:**
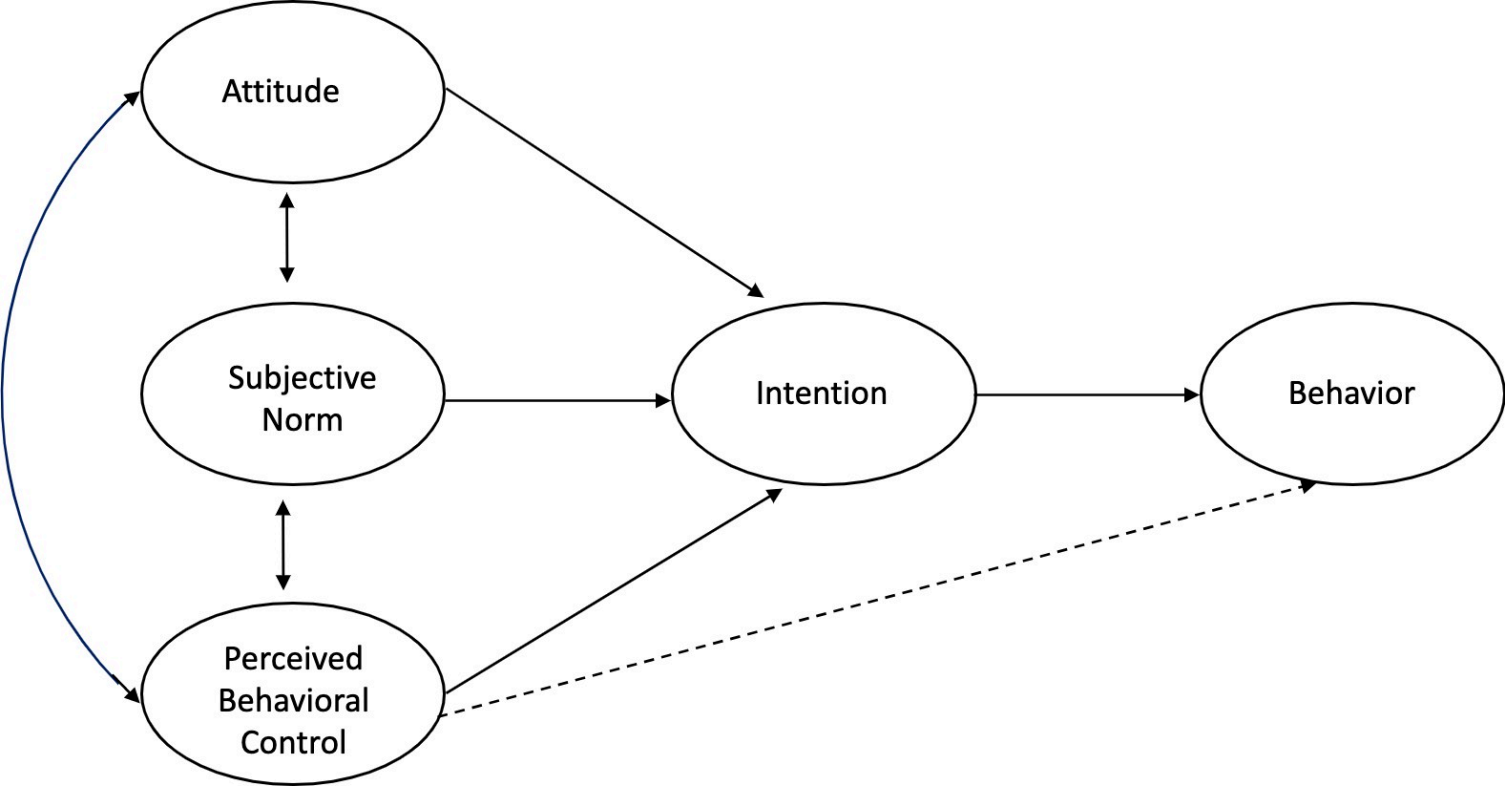
Theory of planned behavior.

The theory of planned behavior assumes that behavior is rational and proposes that behavioral intention directly influences behavior (Fig 1). Behavioral intent is affected by three constructs: attitude, subjective norm, and perceived behavioral control. Attitude is conceptualized as a person’s favorable or unfavorable perception of a behavior. Subjective norm is conceptualized as an individual’s perceptions of their peers’ approval or disapproval of a behavior. Perceived behavioral control is conceptualized as an individual’s perception of their ability to perform a behavior and that they can control their behavior. In order for behavioral *intention* to translate to *actual* behavior; an individual needs to have the ability to perform a behavior. Therefore, it is hypothesized that perceived behavioral control directly influences actual behavior. A review of TPB research has shown consistent patterns among TPB constructs across a variety of contexts. Behavioral intent has been shown to be a strong predictor of behavior, attitudes have been shown to a strong predictor of behavioral intent, social norms have often shown a weak association with behavioral intent, and perceived behavioral control has shown a moderate association with both behavioral intent and behavior [32].

Conceptually, the TPB is well suited to explain instructors’ use of RBIS after participating in professional development. The theory accounts for instructor attitudes, social norms, and their perceived ability (knowledge and skill) to use RBIS. Positive attitudes about RBIS have been described as a pre-condition to increasing RBIS use [30], while negative attitudes have been identified as a barrier to RBIS use [17,24]. RBIS skill and knowledge (perceived behavioral control) have been shown to be positively related to instructors’ use of RBIS [14]. Fundamentally, the goal of instruction-focused professional development is to “develop expert competence” and skill in using and thus adopting new teaching practices [9 p. 6]. Social norms (departmental culture and support) have been identified as a barrier to instructors’ use of RBIS when they perceive their department to be unsupportive of RBIS, and as a support to using RBIS when they perceive their department to be supportive [16,18,24–28].

The TPB is frequently modified to account for context-specific factors that are not included in the core theory, but are known or suspected to influence the target behavior [32]. Here, this flexibility accommodates findings from prior research that has identified individual, institutional, and teaching context factors that can affect an instructors’ intent or motivation to implement RBIS. For example, research on RBIS use has shown that, class sizes, prior experience, and course coordination have been linked to RBIS use [16,22,29,38–42].

In applying the theory to this study, the behavior of interest is instructors’ frequency of use of IBL teaching practices. We considered IBL workshops as PD that can influence instructors’ attitudes about IBL and perceived behavior control (e.g., IBL knowledge and skill) which subsequently influence their frequency of use of IBL teaching practices. Individual characteristics, including IBL experience, and local teaching contexts (support from department colleagues, class size and course coordination) can also affect the degree to which instructors plan to implement IBL or actually implement IBL teaching strategies.

### Practical context for the study: IBL intensive workshops

The setting for the research was a professional development workshop for college mathematics instructors on inquiry-based learning. Here we offer a brief description of IBL as a teaching approach and then describe the workshops in which study subjects participated. IBL is a classroom approach used in mathematics that is consistent with research on learning and has been linked to evidence of positive student outcomes [43–45]. Laursen and Rasmussen [46] describe four pillars of inquiry-based mathematics education that emphasize students’ individual work and collaborative sense-making around meaningful mathematical challenges, and instructor design and facilitation choices that draw out and build on student ideas and that attend to equity in students’ learning experiences. IBL teaching can be broadly characterized as highly interactive and features teaching practices such as student presentations, small group discussions, and/or whole-class discussion. Lecture is deemphasized, although instructors may utilize lectures sparingly to signpost, summarize and recap class activities. Use of IBL teaching practices is variable and there is not a prescribed way to use them; instructors can employ some or all of these practices and use some more or less frequently. A growing number of undergraduate mathematics instructors are using IBL teaching practices, many of whom have participated in an IBL teaching-focused workshop.

In this study, we draw on data from instructors who took part in one of 22 multi-day IBL workshops that we studied as evaluators. Since 2010, these intensive workshops on IBL have served approximately 700 college mathematics instructors. The workshops sought to increase instructors’ knowledge and skills to implement IBL in their own classrooms, to strengthen their confidence, and to support their IBL decision-making. Four-day workshops were held in the summer at locations around the US and included at least 30 hours of contact time and ongoing follow-up support.

While the workshop hosts and leaders varied somewhat over time, broad features of the workshops were consistent, especially the use of active and collaborative learning methods for teaching instructors about IBL. The current workshop model, used in 18 of the 22 workshops, is grounded in research and practical experience with PD and incorporates video lesson study, educational research, IBL facilitation skills, and personal work time [47]. Exposure to educational research can help to strengthen participants’ attitudes about the effectiveness of IBL methods, while video lesson study and sessions on facilitation skills strengthen their IBL knowledge and skill. Personal work time allows participants to begin the implementation process by planning how they will implement IBL practices in a specific course. These workshops accommodate instructors’ diverse teaching settings because they do not promote a particular curriculum or classroom practice, but rather focus on pedagogical principles that can be applied in a variety of courses and classroom settings [46]. Workshop participants are also invited to participate in ongoing follow-up support in the form of an ongoing email list, where they can discuss implementation of IBL teaching practices and address challenges, barriers, and aids to implementation. The workshops have been described in detail by [47–50].

### Research questions

The purpose of this research was to investigate the linkage between professional development and mathematics instructors’ adoption of IBL teaching practices and to explain this linkage using the theory of planned behavior. To this end, this study addresses the following research questions (RQ):

1. To what degree do mathematics instructors’ IBL attitudes, knowledge, and skill change after professional development?
2. How do teaching practices change 18 months after professional development?
3. What is the relationship between teaching practice, professional development, and other factors (e.g., individual, institutional, and teaching contexts)?

To answer Research Question 1, we examined changes over time in instructors’ knowledge, skills, and attitudes related to IBL practice, which the workshop was intended to influence. To answer Research Question 2, we examined changes over time in a specific list of teaching practices that prior work has previously connected to IBL approaches and that were expected to change as a result of workshop participation.

Lastly, Research Question 3 concerns how workshop participation and other factors are related to the use of these new teaching practices after professional development. We explored RQ3 by applying the Theory of Planned Behavior to construct a statistical model from the set of workshop outcome measures, considering differences in participants’ individual characteristics and their institutional and teaching settings.

## Methods

### Data collection and sample

Workshops were nationally advertised and participants self-selected by applying to participate; they were admitted by the workshop organizers in application order. Workshops typically included 25–40 participants. Given the workshops’ length (~4 days), they were highly motivated to attend and supported to do so by project grants and/or institutional finaing. This research was approved by the University of Colorado Boulder Institutional Review Board under the exempt category for project numbers 15–0262, 12–0441, and approved under the expedited category for project number 0809.20. Written consent was obtained from survey participants.

Participants completed a pre-workshop survey about one month before their workshop, a post-workshop survey immediately after, and a follow-up survey about 18 months later. The pre-workshop survey was administered one month prior to the workshop, to provide information about workshop participants’ backgrounds and teaching plans to workshop facilitators. Analyses were limited to participants from the 2010–2019 workshops (*n* = 684) who completed the pre-workshop, post-workshop and 18-month follow-up surveys (*n* = 361), yielding a 60% completion rate.

About half of the participants who completed all three surveys (49%) had some prior IBL teaching experience. The sample included instructors from a range of institution types: Ph.D.-granting 26%, Master’s-granting 24%, four-year 44%, two-year 6%. Half of the participants were early-career instructors with five years or less teaching experience (50%), including people in tenured, tenurable, and non-tenure-track positions. Over half (56%) were women, higher than the general representation of women in mathematics. Most identified their race as white (95%) and a few identified their ethnicity as Latino/a (3%).

### Measures

Surveys included well-established measures [48–50] from several categories related to our theoretical framework (TPB), including measures of professional development outcomes, individual characteristics, institutional characteristics, and teaching contexts. Some items were used in evaluation to measure changes of interest to the workshop leaders, and in doing so collected data not used in this study. In this section, we describe these measures in each of these categories as they relate to measuring change over time for RQs 1–2, and to modeling the TPB for RQ3.

#### Professional development outcomes and teaching practices

Four measures were hypothesized to be related to workshop participation outcomes. They were reflective of TPB constructs and used to answer all three research questions. First, “behavior” as identified in the TPB was derived from measures of self-reported teaching practices in a specific course that the workshop participant chose as their target course and worked on during the IBL workshop. We asked participants on the pre-workshop survey and follow-up survey to indicate their frequency of use of 11 teaching practices using the following scale: 0 = never, 1 = once or twice during the term, 2 = about once a month, 3 = about twice a month, 4 = weekly, 5 = more than once a week, or 6 = every class.

As described in Hayward et al. [51], five of the 11 teaching practices are classified as ‘core IBL’ practices because they characterize all variations of IBL that were emphasized in workshops: decreased use of instructor activities, including lecture and instructor problem-solving on the board, and increased use of student activities, especially student presentations of their own work and student discussion in small groups or as a whole class. Using these five, we created a composite variable that we call IBL intensity, as an overall measure of instructors’ use of IBL methods. IBL frequency scores were calculated for pre workshop and follow-up time points and are based on the frequency values of the five core teaching practices as follows:

$$\begin{array}{c} IBL\;frequency=student\;group\;work+student\;presentation+class\;discussion\\ ‐lecture‐instructor\;solving\;problems. \end{array}$$

Thus, the IBL frequency score is used to answer RQ2 and, for RQ3, as the measure of “behavior” for the TPB. Scores ranged from -12 to 17; higher scores indicate more frequent use of IBL teaching practices and less frequent use of instructor lecture and instructor problem solving. Both pre-workshop and follow-up IBL frequency scores showed acceptable, but marginal, internal consistency for the subset of IBL core practices that were expected to increase after workshop participation (α = 0.56, α = 0.58, respectively). The relatively low internal consistency of pre-workshop and follow-up scores can be attributed to the variability in IBL teaching, as the workshops purposefully espoused a “big tent” approach to accommodate instructors’ preferences, student audiences, and course and institutional contexts [51]. Consistency was slightly better for practices that were expected to decrease after workshop participation; both pre-workshop and follow-up IBL intensity scores were acceptable (α = 0.81, α = 0.78, respectively).

Three items on the post-workshop survey represented the latent variable “intent” in the TPB (RQ3) and showed acceptable internal consistency (α = 0.70). We asked participants **“**How likely are you to implement IBL in the coming academic year?” and **“**If not this year, how likely are you to implement IBL in the following academic year?”. Both items used the same five-point scale (1 = Not at all likely, 2 = Somewhat unlikely, 3 = Somewhat likely, 4 = Rather likely, 5 = Definitely). Another item asked, “How motivated do you feel to incorporate inquiry into your teaching methods?” and used a four-point scale (1 = None, 2 = A little, 3 = Some, and 4 = A lot).

Instructors’ beliefs about the effectiveness of IBL, knowledge of IBL methods, and skill in using IBL methods were measured three times: before the workshop, immediately after the workshop and 18 months after the workshop. These items were used to answer RQ1 and also for “attitude” and “perceived behavioral control” in the TPB model (RQ3), as follows.

For RQ3, “attitude” was defined as participants’ belief in the effectiveness of IBL specifically at the follow-up time point, reflecting their view at the completion of professional development. Participants were asked “To what extent do you believe inquiry-based strategies are an effective learning method?” and rated this item on a four-point scale (1 = Don’t know, 2 = Not very effective, 3 = Somewhat effective, 4 = Highly effective).

The latent variable “perceived behavioral control” in the TPB (RQ3) used participants’ ratings for both knowledge of IBL methods and skill in using IBL methods, again only at the follow-up time point. The “knowledge” item asked respondents to rate their current level of skill in inquiry-based teaching and the “skill” item asked to rate their current level of knowledge of inquiry-based learning in math education. Both items used the same four-point scale (1 = None, 2 = A little, 3 = Some, and 4 = A lot) and showed acceptable internal consistency (α = 0.66).

#### Individual, institutional, and teaching context measures

The pre-workshop and follow-up surveys asked about individual and institutional characteristics as well as teaching context, and these data were used in the following ways.

The fifth TPB construct in the model used to address RQ3, “subjective norm,” was a latent variable derived from two follow-up survey items related to collegial support that were validated in prior research [27]. On follow-up surveys, respondents were asked to rate “Support from your colleagues in the department to use IBL in your teaching” and separately, “Support from your department head or chair to use IBL in your teaching.” Both items used the same four-point scale (1 = Not at all supportive, 2 = Mostly not supportive, 3 = Mixed or moderate support, 4 = Mostly supportive) and showed acceptable internal consistency (α = 0.78).

Some individual characteristics were reported on pre-workshop surveys and included career stage, years of teaching experience, gender, ethnicity, race, and prior experience with IBL methods. Pre-workshop surveys also included measures of institutional characteristics including institution type (by highest math degree awarded) and minority-serving status. Additional measures of teaching context were reported on the follow-up survey to reflect some elements of the particular context of respondents’ IBL use rather than their general teaching context. These included class size, student majors, student level (e.g., first-year), course subject (e.g., calculus I), and information about whether the course they taught was coordinated in any way, such as by sharing syllabi, exams, or assessments across multiple sections of a course.

In a series of preliminary analyses (e.g., ANOVA, regression) we checked for differences in both intent to implement IBL and IBL frequency, and changes in IBL frequency, by all individual characteristics, institutional characteristics, and teaching contextual factors that did not correspond to any TPB construct. These analyses yielded few statistically significant differences when controlling for all other factors. Three tests did show statistically significant differences: participants with prior IBL experience (individual characteristic) showed higher intent to implement IBL, and participants who taught classes with small numbers of students, and those who taught a coordinated course, had higher IBL intensity. To maximize model parsimony, only these three variables (prior IBL experience, small class size, and course coordination) were included in the structural equation model used to answer Research Question 3. They were measured as follows:

- Prior experience with IBL was a dichotomous measure, “Have you ever taught an IBL class before?” (yes = 1, no = 0).
- Class size was measured by one item asking: “How many students are typically in this course?” and included four response options: 25 or less, 26 to 35, 36 to 50, more than 50. Preliminary data analyses showed that instructors who taught classes with 25 or less had use IBL methods more frequently than all other class size categories and found no differences in IBL frequency between any of the three larger class size categories. Therefore, these options were collapsed into two categories: respondents who taught a class with 25 or less students and respondents who taught classes with over 25 students.
- Course coordination was measured by one item that asked respondents to select multiple types of course coordination: “Do you coordinate with others for this course?” and included five responses (e.g. sharing course materials, common exams) derived from prior research on course coordination in calculus [40]. A dichotomous variable was created that treated any response indicating one or more types of coordination as having taught a coordinated course, and otherwise a non-coordinated course.

In sum, intent to implement and the three factors posited by the theory to directly affect that behavioral intent were all measured at the completion of professional development. Teaching behaviors reflecting implementation of IBL in a specific mathematics course were measured 18 months later, as were characteristics of the targeted course. General individual and institutional characteristics for each participant were measured prior to the workshop and incorporated into the model after initial testing.

### Data analysis

To answer Research Question 1, we used SPSS (SPSS 26, Inc., Chicago, IL, USA) to conduct a one-way ANOVA with repeated measures to check for differences in IBL attitudes, knowledge, and skill over the three survey time points. ANOVA is a robust statistical procedure that can be used with ordinal four-point items [52]. One measure, post-workshop IBL attitude, showed skewness and Kurtosis values outside of acceptable ranges [53]. Therefore, for this comparison we also conducted a Kruskal-Wallis test of means for each pair of time points for IB: attitude. Kruskal-Wallis test is a non-parametric test that can be used to test for differences in means of non-normal dependent variables. This analysis was limited to respondents who reported their IBL knowledge, skill, and attitude at the pre-workshop, post-workshop and 18-month follow-up surveys (n = 315).

RQ2 was answered by using a paired samples t-test to test for differences in pre-workshop and follow-up IBL intensity scores. Both pre-survey and follow-up survey IBL frequency scores met all assumptions of normality. This analysis was limited to respondents who reported teaching practices on both the pre-workshop and 18-month follow-up surveys (n = 254).

A structural equation model was used to answer Research Question 3 (AMOS 17, SPSS Inc., Chicago, IL, USA). This analysis was limited to the 322 survey responses that contained no missing data for all variables included in the model. Mardia’s coefficient was calculated to assess multivariate normality and our data exceeded the recommended maximum value of 3.0. Therefore, bootstrapping estimation was used to assess model fit and calculate model estimates [54]. Bootstrapping was also used to test the significance of indirect effects in the structural model. To assess model fit using bootstrapping estimation, Bollen-Stine probability values ≤ 0.05 indicate acceptable model fit [54]. We also used maximum likelihood estimation to provide model fit indices that may be more familiar to readers. Two fit indices (CFI and RMSEA) were used to assess model fit for maximum likelihood estimation. CFI values range from 0–1, where values ≥ 0.95 are considered well-fitting [55]. RMSEA values ≤ 0.05 indicate close fit [56]. Together, the model fit indices and parameter estimates were used to determine how well the measurement and structural models fit our data.

A two-stage modeling approach was used. First a confirmatory factor analysis was performed to assess the convergent and discriminant validity of three latent factors: social norm, perceived behavioral control, and behavioral intent. In stage 2, a structural model tested the TPB using both latent and observed variables and included three additional observed personal and contextual variables outside of the core model. As discussed in the ‘Measures’ section, these variables included prior IBL teaching experience, small class size, and course coordination.

## Results

First, we describe findings related to RQ1, addressing the degree to which IBL attitude, knowledge, and skill change after professional development. A one-way ANOVA with repeated measures was conducted to test for differences in IBL attitudes, IBL knowledge, and IBL skill across three time points (pre-workshop, post-workshop, and follow-up survey).

This analysis showed statistically significant increases in IBL attitude, knowledge, and skill from pre to post workshop (Table 1). Effect sizes (*η*^2^) indicate the largest increases were in IBL knowledge and skill, with a smaller positive change in attitude about IBL effectiveness. From post-workshop to 18-month follow-up, there was no difference in knowledge; a statistically significant, albeit minimal, decrease in attitude; and a statistically significant increase in skill. Kruskal-Wallis tests confirmed the results of the ANOVA (see Table 1). Overall, workshop participants made significant gains in IBL attitude, knowledge, and skill, after participating in professional development, and these gains were sustained 18 months after the workshop.

**Table 1:** Changes in IBL attitude, knowledge, and skill: ANOVA results for differences in mean scores by time point (n = 315).

	Pre-workshop		Post-workshop		18-month Follow-up		Omnibus Statistics		
IBL measure	*M*	*SD*	*M*	*SD*	*M*	*SD*	*F (*df = 2, 313)	*p*	*η* ^2^
Attitude	**3.29**	0.89	**3.85**	0.38	**3.70**	0.51	72.92	<0.001	0.23
Knowledge	**2.44**	0.71	**3.30** ^#^	0.62	**3.20** ^#^	0.60	250.94	<0.001	0.47
Skill	**1.95**	0.73	**2.57**	0.64	**2.82**	0.60	254.85	<0.001	0.47

Note. *M* = mean, *SD* = standard deviation, *F* = *F* statistic, p = probability, *df* = degree of freedom, *η*^2^ = partial eta squared (effect size). Means within each row differ at p < 0.001 except for those marked with #.

**Fig 2:**
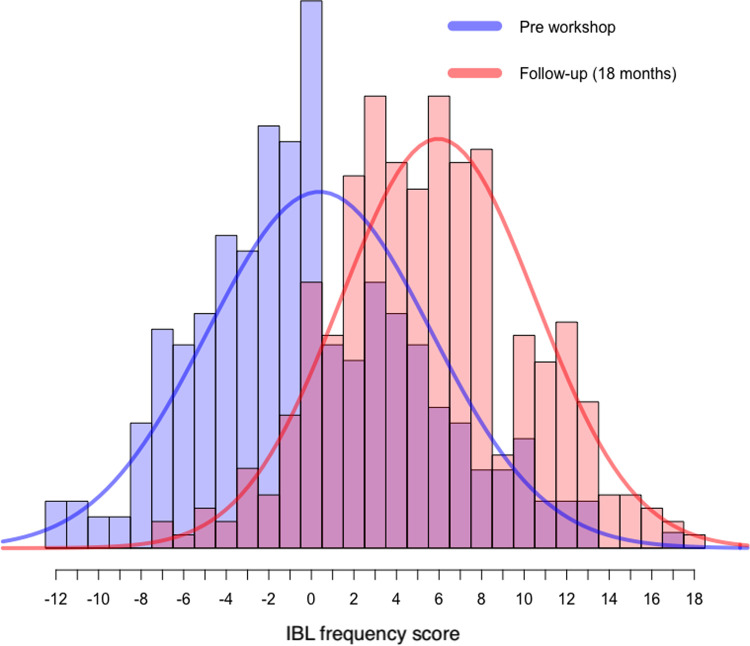
Change in IBL teaching practice: Distributions of IBL frequency scores before workshop and at follow-up.

To answer RQ2, the degree to which teaching practices change after participating in professional development, we used a paired samples t-test (*n* = 254) to test for differences between pre-workshop and follow-up IBL frequency scores (Fig 2). Follow-up scores (*M* = 5.32, *SD* = 4.52) were significantly higher (*t*(230) = 14.15, *p* < 0.001), than pre-workshop scores (*M* = 0.05, *SD* = 5.21). A large effect size (*Cohen’s d* = 0.88) indicated a strong positive change in IBL teaching frequency from pre-workshop to follow-up.

Structural equation modeling was used to answer RQ3. The measurement model showed acceptable fit for maximum likelihood estimation (CMIN  =  (df) 15.126 (12) *p = 0*.*235*, CFI  =  0.99, RMSEA  = 0 .028) and bootstrapping estimation (B-S *p = 0*.*234)*. As shown in Table 2, all factor loadings were positive and had significant standardized regression weights above 0.03 with their related latent variables, indicating convergent validity. Latent factor correlations less than 0.40 indicated discriminant validity. Following the acceptable model fit indices and regression weights from the measurement model in stage 1, we proceeded to performing a structural model in stage 2.

**Table 2:** Standardized factor loadings for TPB constructs.

Latent variable	Observed variable	ML estimates	Bootstrap estimates
Social norm	Collegial support	0.78***	0.78***
	Chair support	0.81***	0.81***
Perceived behavioral control	IBL knowledge	0.70***	0.70***
	IBL skill	0.88***	0.88***
Intent	Intent to implement in coming year	0.73***	0.73***
	Intent to implement in following year	0.65***	0.65***
	Motivation to use IBL	0.63***	0.63***

* p<0.05

** p<0.01

*** p<0.001.

**Fig 3:**
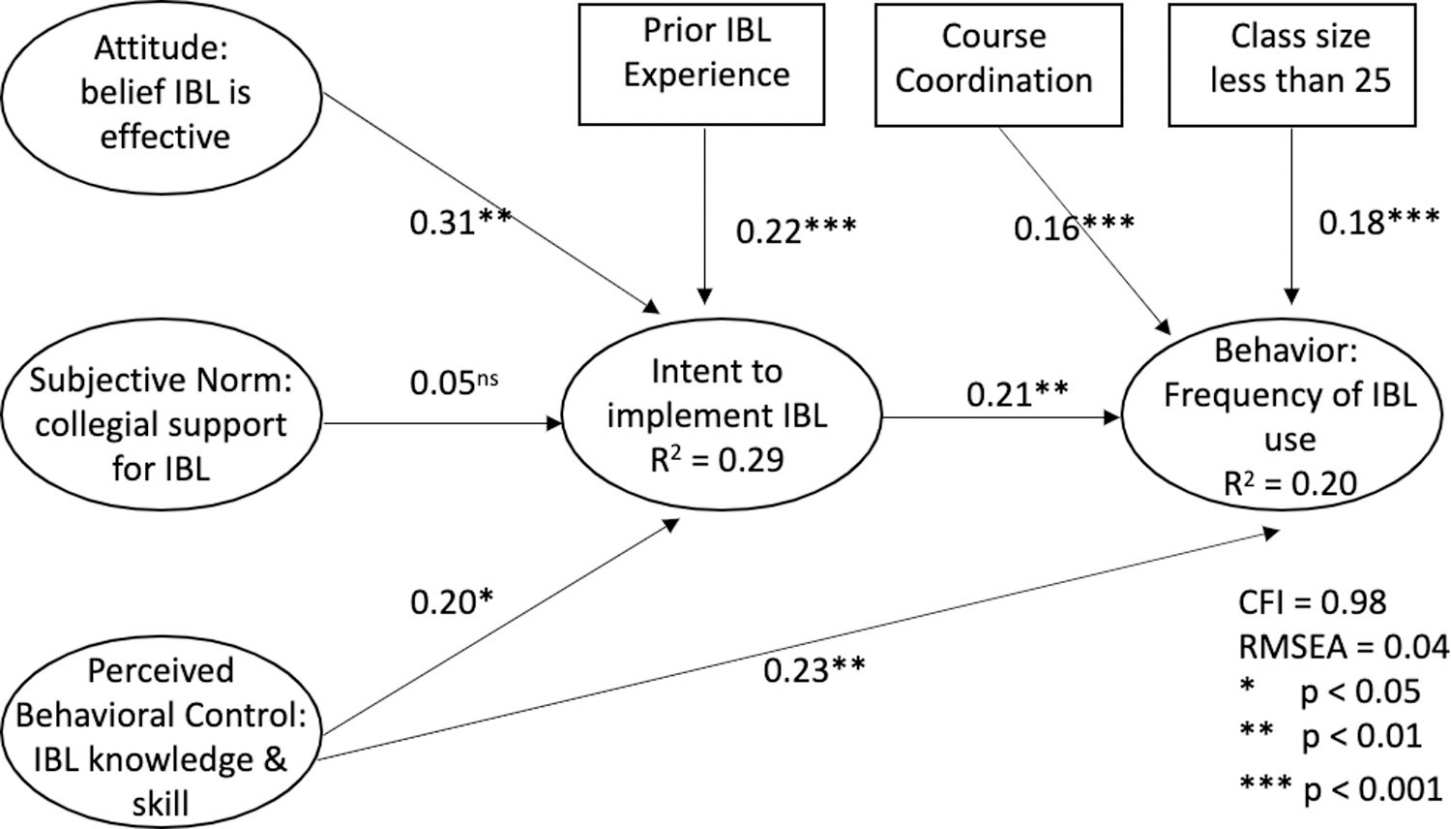
Structural equation model of the theory of planned behavior with standardized direct effects.

Descriptive statistics for all variables included in the structural model are shown in Table 3. The structural model (Fig 3) showed acceptable model fit for maximum likelihood estimation CMIN  =  (df) 63.718 (46) *p = 0*.*043* (*CFI* = 0.8, *RMSEA* = 0.4) and bootstrapping estimation (B-S = *p = 0*.*234*). The structural model explained a moderate amount of variability in intent to implement IBL (*r*^2^ = 0.29) and in IBL intensity (*r*^2^ = 0.20).

**Table 3:** Descriptive statistics for all variables included in the structural equation model (n = 322).

TPB construct	Observed variable	Mean	SD
Attitude	Belief in the effectiveness of IBL	3.68	0.54
Social norm	Collegial support	3.41	0.71
	Chair support	3.55	0.68
Perceived behavioral control	IBL knowledge	3.20	0.60
	IBL skill	2.81	0.65
Intent	Intent to implement in coming year	4.62	0.69
	Intent to implement in following year	4.79	0.38
	Motivation to use IBL	3.86	0.37
Behavior	IBL frequency	5.52	4.57
Factors outside of TPB	Prior IBL experience	50% (n = 161)	
	Course coordination	25% (n = 81)	
	Class size of 25 students or less	47% (n = 151)	

As shown in Table 4, we found positive, statistically significant standardized direct effects in all but one of the specified paths in the structural model. Of the four variables theorized to influence intent to implement IBL, IBL attitude had the strongest effect, while prior IBL experience and perceived behavioral control showed similar moderate effects. Lastly, subjective norm (collegial support), had a minimal non-significant direct effect on intent to implement IBL.

**Table 4:** Standardized direct and indirect effects of the structural model.

Direct effects on Intent	ML estimates	Bootstrap estimates
Attitude	0.31***	0.31**
Social norm	0.05^ns^	0.05^ns^
Perceived behavioral control	0.20**	0.20*
Prior IBL experience	0.22***	0.22***
Direct effects on behavior		
Intent	0.21***	0.21**
Perceived behavioral control	0.23***	0.23**
Class size	0.18***	0.18***
Course coordination	0.16***	0.16**
Indirect effects on behavior		
Attitude	0.07	0.07**
Social norm	0.01	0.01^ns^
Perceived behavioral control	0.04	0.04*
Prior IBL experience	0.05	0.05**

*p<0.05

**p<0.01

***p<0.001.

Four variables were predicted to directly affect behavior (frequency of IBL use), and all had similar moderate positive associations with behavior: perceived behavioral control, behavioral intent (intent to implement IBL), class size, and course coordination. Four variables were also predicted to have indirect effects on behavior. Social norms had a nonsignificant indirect effect on behavior, while attitude, perceived behavioral control and prior IBL experience all showed similar and minimal indirect effects.

Fig 3 summarizes these results graphically. In summary, instructors’ intention to use IBL methods was directly affected by their post-workshop reports of their attitude about IBL, their perceived behavior control to use IBL methods (skill and knowledge,) and prior experience using IBL. Instructors’ frequency of use of IBL methods was directly affected by their intent to use IBL methods, their capacity to use IBL (knowledge and skill), teaching a class of 25 students or less, and teaching a course coordinated with others. Instructors’ frequency of use of IBL methods was indirectly affected, albeit minimal, by their IBL attitudes, prior IBL experience, and their capacity to use IBL methods (knowledge and skill).

Instructors’ perceived behavioral control to use IBL, i.e. their ratings of their IBL knowledge and skills after participating in a workshop, had the largest total effect on IBL intensity of any factor included in the model. Social norms had no direct effect on instructors’ intent to use IBL methods and had no indirect effect on their intensity of use of IBL methods.

## Discussion

The findings show strong linkages between professional development and use of IBL teaching practices. Findings related to RQ1, the short- and longer-term workshop participants outcomes, showed that participants’ IBL attitudes strengthened positively, and their IBL knowledge and skills increased after participating in professional development. Instructors reported strong positive attitudes about the effectiveness of IBL before participating in professional development, yet these still significantly increased after attending workshops. Their strengthened positive attitudes persisted for 18 months after the workshop, which indicated the workshop had a lasting effect. Sustained gains in attitudes may be attributed to ongoing implementation support from workshop facilitators and other participants that, other data show [57], helped them work through or avoid experiences that could have otherwise diminished their attitudes about the effectiveness of IBL.

By comparison to attitude, instructors reported large gains in IBL knowledge and skill from pre- workshop to post-workshop that were also sustained 18 months after the workshop. Gains in IBL knowledge occurred during the workshop, while gains in skills occurred both during the workshop and during the 18-month follow-up period. Knowledge is gained quickly in a workshop, while skills develop over time with practice. These findings are consistent with prior research that showed similar levels of gains for attitudes, knowledge, and skill after PD workshop participation [14]. Such strengthened positive attitudes, knowledge and skills may stem from the workshops’ emphases on educational research about IBL teaching and on design principles and practical ideas for IBL courses [47].

Findings related to RQ2, the degree to which instructor teaching practices changed after participating in PD, instructors’ teaching became more IBL-intensive and less instructor-focused 18 months after participating in PD. The large effect size of 0.88 indicated a substantial shift in IBL teaching intensity. It seems instructors were not merely experimenting with IBL methods but making substantial shifts in their teaching. These findings are consistent with prior research that has shown high initial adoption of RBIS following professional development [13,14], although other research has shown that many instructors abandon RBIS soon after initial adoption [58,59]. We suggest that IBL workshops aid implementation by allowing instructors time to plan their course and adapt methods to their own context, and by providing ongoing follow-up support [47,50].

The following sections are related to RQ3 and provide a discussion of the model as whole, followed by a discussion of each relationship in the model to reveal how workshop participation, and other contextual factors are related to RBIS use. Overall, findings related to RQ3 were generally consistent with the TPB and with prior research. The structural model explained 29% of the variability of instructors’ intent to use IBL methods and 20% of their self-report use of IBL methods, which is consistent with previous validations of the TPB [32]. The structural model specified four factors directly related to intent to implement IBL, four factors directly related to IBL intensity, and four factors indirectly related to IBL intensity. Only one factor, social norms, did not have statistically significant effects. Overall, the amount of variance explained by the model and the positive statistically significant relationships together indicate that the TPB fit our data as effectively as other diverse data and contexts [32]. The following sections provide a discussion of each relationship in the model to explain how workshop participation and other contextual factors are related to RBIS use.

Perceived behavioral control had moderate direct effect on behavioral intent, and moderate direct and minimal indirect effect on behavior. These findings indicate that the greater instructors’ level of IBL knowledge and skill, the more likely they were to intend and intensively use IBL methods. Knowledge and skills gained in the workshop were the strongest predictor of intensity of IBL use, and this in turn suggests that participation in professional development is more influential in determining teaching practice than any individual, institutional, or teaching context factor included in the model.

Instructors’ attitudes about the effectiveness of IBL had a strong direct effect on intent to implement IBL and a minimal indirect effect on instructors’ intensity of use of IBL methods. These findings indicate that the more positive attitudes instructors hold about the effectiveness of IBL, the more likely they are to plan to implement IBL methods. Prior research has found that faculty beliefs regarding the positive results of RBIS are the best predictor of faculty use of RBIS [60], and we likewise find instructors’ attitudes to be also highly predictive of their intent to use RBIS and subsequently their actual use of RBIS. Recent works has also highlighted how exposure to educational research can help to strengthen instructors’ attitudes about the effectiveness of RBIS [61].

Instructors’ prior IBL experience had a positive direct effect on their intent to implement and a positive, though minimal, indirect effect on IBL intensity. That is, instructors with prior experience with IBL had a stronger intent to implement and reported more frequent IBL use than those who had no experience. Prior IBL experience has been identified as a factor that aided IBL implementation [29] and, more generally, instructors’ prior experience with RBIS is positively related to RBIS use across STEM disciplines [22].

Remarkably, no other individual or institutional characteristics that we measured rose to statistical significance as influences on intent or implementation. This is striking given participants’ demographic diversity and the range of their teaching experience and situations, from two-year, four-year, masters’ and PhD-granting institutions. In other work, demographic and institutional factors have been thought to be important in adoption of RBIS [17,22,24].

Social norm showed no direct effect on behavioral intent or indirect effect on behavior, indicating that instructors’ perceptions of support from colleagues had no measurable impact on their intent to use IBL nor their intensity of use of IBL methods. This finding is inconsistent with prior research that has shown linkages between department culture or support and RBIS use [16,62] However, this finding is consistent with prior research using the TPB, where social norms typically have weak associations to behavioral intent [32]. One explanation for this finding is that many of the workshop participants received financial support from their department or institution to attend the conference, thus were less likely to perceive lack of support as barrier to RBIS use.

The strong positive relationship between behavioral intent and behavior indicated that instructors with high intent tend to use IBL more frequently than those with weaker intent. This finding is consistent with prior TPB research that has found behavioral intent to be a strong predictor of behavior [32]. In this study we observed that behavioral intent is strongly related to instructors’ use of IBL methods, but is mediated by their perceived ability to use IBL methods and by contextual factors outside of the model. That is, the choice of teaching practice is not just a function of individual choice and capability, but is also constrained or aided by contextual factors [63]. This finding is consistent with other applications of the TPB, which has shown other factors also can aid or inhibit the ones behavior [32].

Two contextual factors, small class size and course coordination, both had similar effects on frequency of IBL use. The moderate positive direct effect of course coordination on IBL intensity indicates that instructors who implemented IBL in coordinated courses used IBL methods more frequently than instructors who worked on their own. This finding is consistent with prior work linking course coordination to use of active learning strategies [38,39]. Course coordination often involves common use of assessments, materials, and teaching practices, and can include meetings where faculty meet to discuss and plan instruction. In the best cases, instructor teams are focused on improving the course over time, often with a focus on the use of RBIS [38,41,42]. And course coordination can also provide supports that overcome barriers to RBIS use, such as supplying ready-to-go active learning materials, sharing strategies and advice to implement them, and promoting department norms that are supportive of RBIS use. Instructors can work collectively to address student resistance and course coverage concerns, and course coordinators are often good sources of professional development, collegial encouragement, and trouble-shooting [38,41,42].

A moderate positive direct effect of class size on IBL intensity indicates that instructors who applied IBL in smaller classes used IBL more intensively within the first year than those who taught larger classes. Some IBL teaching practices (e.g., student presentations) are harder to use in larger classes, especially for new users, and class size has been identified as one of the primary factors making IBL implementation more difficult for new users [29]. Ferrare and Hora [63] also found large classes to be a barrier to RBIS use. However, our finding reflects initial implementation in the first academic year after the workshop and does not speak to whether small classes are important for long-term use of IBL; it is possible, for example, that our data reflect new IBL users’ common and wise choice of small classes as starting points.

Connecting findings from RQ1 and RQ2, it appears that workshops increase IBL capacity, which subsequently increases instructors’ use of IBL teaching practices. Findings about RQ3 support this conclusion, as IBL knowledge and skill had the strongest effect on IBL teaching frequency in this model. A positive attitude about IBL teaching, IBL knowledge and skill, and prior IBL experience were important in supporting instructors’ intentions to use IBL teaching methods, but skills and knowledge also enabled instructors to actually implement IBL. However, workshop participation alone does not fully account for instructors’ use of IBL methods, as teaching contexts can also support or inhibit IBL use. Coordinated courses and small class sizes can aid implementation, while siloed teaching and larger class sizes can inhibit IBL use, despite an instructor’s intention and capacity to use RBIS methods.

These findings point to several practical implications about strategies to advance the uptake of research-based approaches to teaching in STEM higher education. Investments by departments, professional societies, and funders should focus on intensive PD to strengthen instructors’ attitudes about the effectiveness of RBIS and to support development of the knowledge and skill that enable them to effectively use RBIS. This study examined intensive, discipline-based workshops on a particular pedagogical approach; from detailed evaluation data, it is clear that the duration, intensity, workshop design and skilled facilitation all contribute to its impact. Indeed, separate results from our group show that shorter, introductory workshops (1–8 hr duration) led by the same facilitators can generate awareness and interest but do not yield the same degree of behavior change as do the intensive workshops discussed here [64]. Other mechanisms to strengthen instructors’ RBIS attitudes, knowledge, and skill should also be considered; for example, prior work has highlighted how exposure to discipline-based educational research can help increase instructors’ capacity to use RBIS [61].

Targeting departments as loci of change, efforts to inform and train course coordinators could also hasten uptake of IBL and other RBIS, as course coordinators are responsible for coordinating instruction in multiple sections of a given course [65]. Institutional support for initial implementation with small class sizes or team efforts could ultimately offer more students more learning experiences under research-aligned teaching practices, as instructors develop skills in more forgiving circumstances and then learn to adapt their practices to different teaching contexts [66].

The findings also speak to the utility of social science theory to understand change in STEM instruction. The theory of planned behavior can adequately explain changes in teaching practices behaviors and could be utilized to guide design and explain outcomes and processes of professional development in other STEM disciplines that seek to encourage changes in teaching practices. Use of a common theory of change would enable comparisons across disciplines and could reveal PD features and contextual factors that are common or specific to STEM disciplines. The theory should also be applied to other change initiatives such as departmental or institutional efforts, to understand how these contexts may differ from PD initiatives that occur outside of these contexts.

A limitation of this study is its focus on individual change. The social environment of instructors was incorporated into the TPB as an influence on instructors’ intent and actions, but was not directly addressed by the professional development provided to them. Prior work has shown that department cultures and support are associated with RBIS use. Since the results of this study did not show such an association, future work should address the conceptualization and measurement of social norms and its relationship to instructors’ intent to use RBIS and their actual use of RBIS. Instructors’ ability to influence departmental and disciplinary norms, and their own potential role as change agents (e.g., as course coordinators) could be a target of professional development and other levers for change as well [67]. Finally, the study findings, as well as the workshops on which they are based, are situated within the broader context of US higher education, where professional development on teaching is decentralized and often discipline-based; specific findings may not directly apply to other higher education settings.

## Supporting information

S1 Dataset Research question 1 and 2. (CSV)

S2 Dataset Research question 3. (CSV)
